# Simultaneous Adsorption and Degradation of Cr(VI) and Cd(II) Ions from Aqueous Solution by Silica-Coated Fe^**0**^ Nanoparticles

**DOI:** 10.1155/2013/649503

**Published:** 2013-12-15

**Authors:** Yongchao Li, Hongpu Ma, Bozhi Ren, Tielong Li

**Affiliations:** ^1^School of Civil Engineering, Hunan University of Science and Technology, Xiangtan 411201, China; ^2^College of Environmental Science and Engineering, Nankai University, Tianjin 300071, China

## Abstract

Core-shell silica-coated Fe^0^ nanoparticles (Fe@SiO_2_) were prepared in one-step synthesis by aqueous reduction combined with modified Stöber method. The as-prepared Fe@SiO_2_ were then used for simultaneous removal of Cr(VI) and Cd(II) from aqueous solution. Batch tests indicated that Fe@SiO_2_ exhibited high removal capacity toward Cr(VI) and Cd(II). Cr(VI) was removed by Fe@SiO_2_ through reduction rather than adsorption, while Cd(II) removal was mainly through adsorption. The removal rate increased with increasing initial Fe NPs dose and decreased with increasing initial Cr(VI) and Cd(II) concentrations. Cd(II) adsorption was also strengthened by Cr(VI) reduction with the release of OH^−^. The removals of Cr(VI) and Cd(II) were weakened in the presence of cations or humic acid, as a result of aggregation and less active site of Fe@SiO_2_. Overall, the simply prepared Fe@SiO_2_ were potential material for the heavy metals removed from water.

## 1. Introduction

Most heavy metals are toxic and carcinogenic even at very low concentrations and usually cause a serious threat to the environment and the public health. For example, Cr(VI) is a toxic, carcinogenic substance to human and animals [1]. Contrarily, Cr(III) is relatively nontoxic and an essential nutrient for human [2]. Cd(II) is also a potent carcinogen causing damage to the lungs, kidneys, liver, and reproductive organs [3, 4]. Therefore, US Environmental Protection Agency [5] regulates at least ten metals, including chromium and cadmium, as primary contaminants that must be removed from drinking water. There are various conventional techniques applied for removing heavy metals from water. Electroplating wastewater usually contains various heavy metals as cocontaminants; however, few studies have been reported on simultaneous removal of Cr(VI) and Cd(II) from wastewater.

Among the different treatments for removing heavy metals, adsorption has been developed as a simple, efficient, and cost-effective method. Many of adsorbents such as clays, activated carbon, sewage sludge, and plant parts have been used for heavy metal removal [6–9]. But due to extremely small particle size and large surface area, iron nanoparticles (Fe NPs) with a high adsorption capacity are found to be one of the most effective adsorbents for removing heavy metals [10–12]. Moreover, Fe NPs have shown a high chemical reduction rate on several kinds of contaminants, including toxic metal ions [13–15]. For example, Ponder et al. [13] have found that Fe NPs acting as reductants could chemically reduce Cr(VI) to Cr(III). Besides, Fe NPs can proactively attack contaminant plumes in the source zone and potentially reduce the remediation cost and time substantially. However, the applications of Fe NPs have been hindered by the key technical barrier that the nanoparticles tend to agglomerate and grow to micron scale or larger, thereby rapidly losing their mobility and chemical reactivity [16]. At the same time, Fe NPs easily react with dissolved oxygen and water resulting in a reduction of their chemical reactivity owing to the formation of iron oxide [17]. Therefore, many previous studies have focused on the reactivity enhancement of Fe NPs. Then, resin or carbon-supported Fe NPs [18, 19] and Polymer-coated Fe NPs [20, 21] have been commonly prepared to enhance dispersion and antioxidationof nanoparticles.

Recently, nanoparticles with uniform size distribution and high antioxidation are obtained in virtue of SiO_2_ coating [22]. These core-shell nanomaterials may have an important application in Cd(II) adsorption because cationic Cd(II) ion can readily approach negative charged silica surface as a result of electrostatic attraction [23]. In addition, these nanomaterials may reduce the Cr(VI) pollution because silica surface can strongly bind Fe(III) and Cr(III) via surface complexation [24, 25]. However, such core-shell nanostructures are often generated by complicated processes, that is, the preparation of metal NPs followed by the deposition of the inorganic material around them using appropriate synthetic methods. And surface-coupling agents [26, 27] are often used to control the metal particle size or as the primer to facilitate the SiO_2_ disposition. Hence, it is significant to develop a simple method to coat Fe NPs with SiO_2_ shells.

In our earlier paper, a novel one-step method for the preparation of SiO_2_-coated Fe nanocomposites (Fe@SiO_2_) was described [28]. SiO_2_ shell was directly precipitated on the Fe NPs surface through aqueous reduction combined with modified Stöber process. And the Fe@SiO_2_ were effective in Cr(VI) remediation. In this follow-up work, the relevant mechanism for simultaneous removal behaviors of heavy metals by Fe@SiO_2_ was studied. The specific objectives were (1) synthesis of the Fe@SiO_2_ using one-step method without using any of surface-coupling agents, (2) study of Cr(VI) and Cd(II) simultaneous removal kinetics and mechanism, and (3) investigation of the influence of some experimental parameters on the removals of Cr(VI) and Cd(II), such as pH, iron dose, solution concentration, and coexisting ions.

## 2. Experimental Sections

### 2.1. Materials and Reagents

All the chemicals used in this research were analytical grade or higher and were used as received. And deionized water (DI) water was used throughout the whole experiment.

### 2.2. Preparation of Fe@SiO_2_

In a typical synthesis, 0.0436 g FeCl_3_ was firstly dissolved in 30 mL 70% (v/v) ethanol solution. To this solution, 0.1 mL tetraethylorthosilicate (TEOS) was added for the synthesis of silica, and thus theoretical Fe contents of the finally prepared composite was about 37.5 wt.%. Then freshly prepared KBH_4_ (2.69 mmol·L^−1^) was added dropwise to FeCl_3_ solution at 7 : 1 molar ratio with vigorous stirring. Next, Fe NPs were synthesized in the laboratory via the following reaction [29]:

(1) Fe(H2O)63++3BH4−+3H2O →Fe0(S)+3B(OH)3+10.5H2(g)

Excess borohydride was added to accelerate Fe NPs synthesis and ensure silica formation [28]. After 120 min of reaction, the resulting particles were collected by a magnet and then washed repeatedly with DI water to get rid of the excess chemicals. The whole process was carried out in a nitrogen atmosphere. Pure SiO_2_ was also obtained as described above without FeCl_3_.

### 2.3. Characterization of Fe@SiO_2_

The surface morphologies of the samples were characterized by a TEM (Phillips Tecnai F20). XRD analysis was performed using PhilipsD/Max-2500 diffractometer. Details on TEM and XRD analyses have been described elsewhere [28]. To determine the isoelectric point of Fe NPs, the **ζ**-potential of sample was measured as a function of solution pH using a Zetasizer NanoZS (Malvern Instruments, UK).

### 2.4. Batch Removal/Adsorption Experiments

Stock solutions of Cr(VI) and Cd(II) ions (100 mg·L^−1^) were prepared by dissolving K_2_Cr_2_O_7_ and Cd(NO_3_)_2_ in DI water. Batch experiments were performed in 125 mL glass vials capped with Teflon Mininert valves to minimize the oxidation of Fe NPs. Next, a predetermined quantities of Fe@SiO_2_ were added into 100 mL mixed solution of Cr(VI) and Cd(II) and then placed on a rotary shaker with 180 rpm. At given interval, samples were withdrawn by a 1 mL-syringe, filtered through a filter (0.22 *μ*m), and tested for Cr(VI) and Cd(II) concentration, respectively. In addition, to examine the role of silica shell, an experiment was designed to mix Fe@SiO_2_ with Cr(VI) and Cd(II) independently at the same reaction condition.

To study the effect of initial iron dose on the Cr(VI) and Cd(II) removals using Fe@SiO_2_, 0.1, 0.15, 0.2, and 0.3 mg·L^−1^ Fe NPs were added to the reactor vials, respectively, with initial concentrations of both metal ions at 50 mg·L^−1^ and solution pH at 6. The effects of initial Cr(VI) and Cd(II) concentrations were investigated by varying the Cr(VI) and Cd(II) concentrations from 50 to 80 mg·L^−1^ and reacting with 0.15 g·L^−1^ Fe NPs at pH 6. To examine the effect of initial pH, solutions were adjusted to the desired levels, pH from 4 to 9. The effect of competitive cations (Ca^2+^, Mg^2+^, K^+^, and Na^+^) was investigated by adding 10 mmol·L^−1^ of a cation to the reactor vial, respectively. It was also conducted to investigate the effect of 10 mg·L^−1^ humic acid (HA) on the heavy metal removal. All experiments were performed at room temperature (25°C). To assure data quality, all experiments were performed in duplicate.

### 2.5. Analytical Measurements

The Cr(VI) concentration was tested using the diphenylcarbohydrazide method [30], and the concentration of Cd(II) was measured by an ICP-AES (ICP-optima 2001DV, Perkin-Elmer, USA). The pH was measured throughout the experiments by using a pH meter (PB-10, Sartorius, China).

## 3. Result and Discussion

### 3.1. Characterization

The XRD results demonstrated that the SiO_2_ was produced and adsorbed to Fe NPs. TEM analysis indicated that the synthesized sample had a clearly distinguished core-shell structure: the dark cores were attributed to Fe and the grey shells were attributed to SiO_2_. The mean particle size of Fe NPs was calculated to be 25 nm. And the SiO_2_ shell had series of small pores [28].

**Figure 1:**
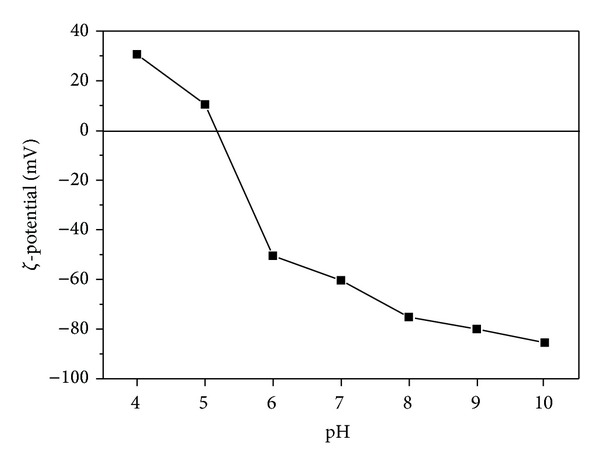
**ζ**-potentials of Fe@SiO_2_ as a function of solution pH.

Figure 1 shows the **ζ**-potential of Fe@SiO_2_ as a function of pH. The **ζ**-potential of Fe@SiO_2_ decreased with the increase of pH. The isoelectric point of bare Fe NPs was about 8~8.5 [31, 32]. However, with the presence of silica shell on Fe NPs surface, a much lower isoelectric point 5.2 was observed for Fe@SiO_2_. It indicated that the prepared Fe@SiO_2_ were more negatively charged.

### 3.2. Reduction of Cr(VI) and Cd(II) Independently Using Fe@SiO_2_

**Figure 2:**
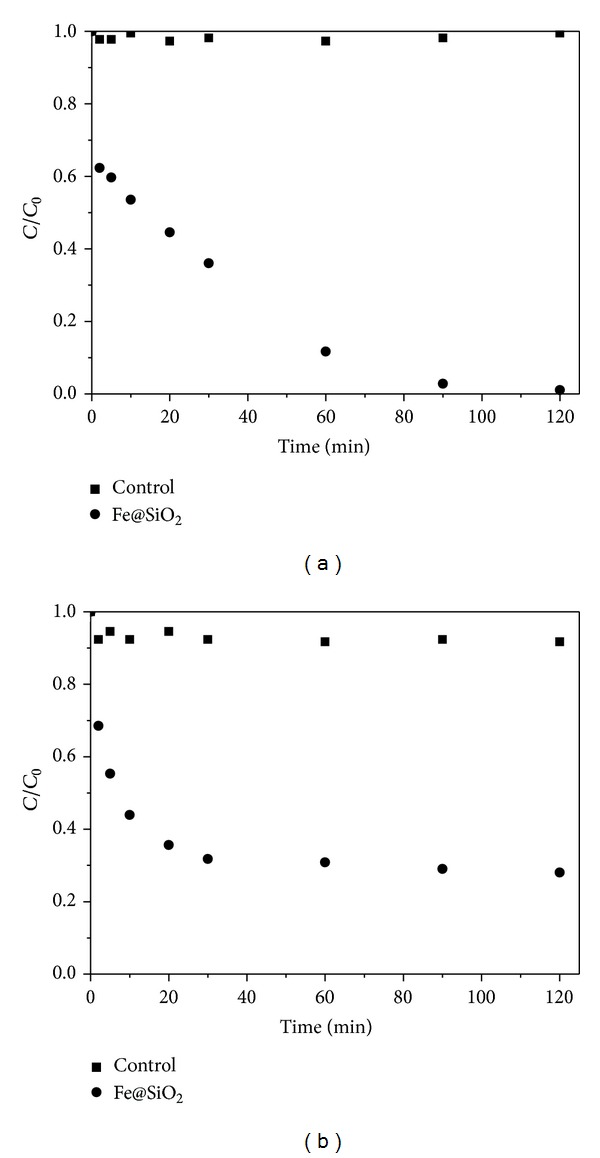
Removal of Cr(VI) (a) and Cd(II) (b) individually using Fe@SiO_2_. Initial Cr(VI) and Cd(II) concentration: 70 mg*·*L^−1^, Fe dose: 0.15 g*·*L^−1^, pH: 6.0 ± 0.1, and temperature: 25°C.

Figure 2(a) shows the removal capacity of Cr(VI) by Fe@SiO_2_ at an Fe dose of 0.15 g·L^−1^ and initial Cr(VI) concentration of 70 mg·L^−1^ and pH of 6.0 ± 0.1. The normalized residual concentration (*C*/*C*_0_) was used to describe the removal rate. After 120 min of contact, almost 100% Cr(VI) was removed by Fe@SiO_2_ and Cr(VI) removal capacity was calculated to be 467 mg Cr/g Fe. The pure SiO_2_ (without Fe NPs) was investigated as a control. The control reactor showed no loss of Cr(VI) during the whole experiment. Compared with that of reported stabilized Fe NPs [13, 17], a significant increase in Cr(VI) removal was obtained. This was because Cr(VI) ions could readily approach small Fe NPs surface by passing through the porous SiO_2_ shell. The XPS analysis in our previous research indicated that Fe was primarily oxidized to Fe(III) and Cr(VI) was reduced to Cr(III) [30]. At the same time, Silica surface can strongly bind Fe(III) and Cr(III) via surface complexation [24, 25]. And the pH of mixed solutions increased to about 9.5 after reaction. Therefore, Fe(III)/Cr(III) hydroxides precipitation on the SiO_2_ shell surface occurred. Obviously, there was an initial sorption phase along with the quick redox reaction during the first 2 min. A similar finding was also obtained by others [33]. This was probably due to the formation of Cr-Fe hydroxides on the Fe^0^ surface.

Similar investigations were conducted for studying the removal efficiency of Cd(II) ions by Fe@SiO_2_. As shown in Figure 2(b), around 72% of Cd(II) was removed by Fe@SiO_2_ in 120 min. pH rose to about 8.4 after reaction because of OH^−^ release when Fe^0^ reacted with H_2_O [34]. Control reactor showed that about 8% Cd(II) was removed by silica. It was calculated that the removal capacities for Cd(II) by Fe@SiO_2_ were approximately 336 mg·g^−1^, while it was only 242 mg·g^−1^ for the uncoated Fe NPs [12]. Therefore, the Cd(II) removal rate was improved greatly by Fe@SiO_2_. As the standard reduction potential of Cd^2+^ (*E*_Cd^2+^/Cd^0^_^0^ = −0.40 V) is very close to that of Fe^2+^ (*E*_Fe^2+^/Fe^0^_^0^ = −0.44 V) and SiO_2_ shell was porous, Cd(II) was removed mainly through adsorption on the Fe NPs surface. The adsorption of Cd(II) on Fe NPs surface was assumed to occur through the following surface reactions [32]:

(2) $$\begin{array}{c} \text{C}{\text{d}}^{2+}+\equiv \text{SOH}⇌\text{SOC}{\text{d}}^{+}+{\text{H}}^{+} \end{array}$$

(3) $$\begin{array}{c} \text{C}{\text{d}}^{2+}+2\left(\equiv \text{SOH}\right)⇌\equiv {\left(\text{SO}\right)}_{2}\text{Cd}+2{\text{H}}^{+} \end{array}$$

(4) $$\begin{array}{c} \text{C}{\text{d}}^{2+}+\equiv \text{SOH}+{\text{H}}_{2}\text{O}⇌\equiv \text{SOCdOH}+2{\text{H}}^{+} \end{array}$$

where ≡SOH represents a surface hydroxyl group. There was also a large amount of silanol on SiO_2_ surface [35]. So, 8% Cd(II) was adsorbed on SiO_2_ surface. In fact, the maximum removal efficiency of Cd(II) ions by Fe@SiO_2_ was achieved within the first 30 min. It might be due to the large amount of adsorptive sites available at the beginning, and the adsorption became slower as the adsorption sites were gradually filled up.

### 3.3. Simultaneous Removal of Cr(VI) and Cd(II) Using Fe@SiO_2_

**Figure 3:**
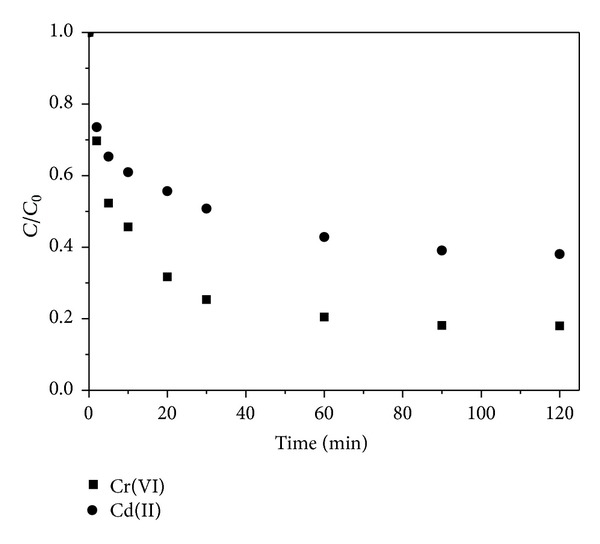
Removal of Cr(VI) and Cd(II) simultaneously using Fe@SiO_2_. Initial Cr(VI) and Cd(II) concentration: 70 mg·L^−1^, Fe dose: 0.15 g·L^−1^, pH: 6.0 ± 0.1, and temperature: 25°C.

The initial pH value of 70 mg·L^−1^ Cr(VI) and Cd(II) mixed solution was around 6. As shown in Figure 3, simultaneous removal of 82% Cr(VI) and 62% Cd(II) by Fe@SiO_2_ was observed after 120 min reaction. And a more efficient removal of Cr(VI) than that of Cd(II) was obtained. This was because the standard reduction potential of Cr(VI) was more positive than that of Cd(II), meaning that Cr(VI) was much easier to be reduced compared with Cd(II) [36]. At the same time, the positive Cd(II) was more easily adsorbed on the negatively charged Fe NPs surface than the negative Cr_2_O_7_^−^ as a result of electrostatic interactions and specific surface bonding [37]. However, in the acidic medium (pH < 7), the speed of Cr(VI) reduction was faster than that of Cd(II) adsorption. As the Cr(VI) reduction reaction proceeded, Fe NPs were dissolved and the active adsorption sites were consequently decreased. Therefore, the adsorption of Cd(II) had declined compared with that of Cd(II) removal individually using Fe@SiO_2_. While the reduction of Cr(VI) with Fe@SiO_2_ was also hindered by the adsorption of Cd(II). In a word, adsorption could be dominated in the removal of Cd(II), while reduction may play a main role in the removal of Cr(VI). According to the ICP-AES analysis, the dissolved iron in water after reaction was only 0.0123 mg.

### 3.4. Effect of Fe NPs Dosage on Cr(VI) and Cd(II) Removal over Fe@SiO_2_

**Figure 4:**
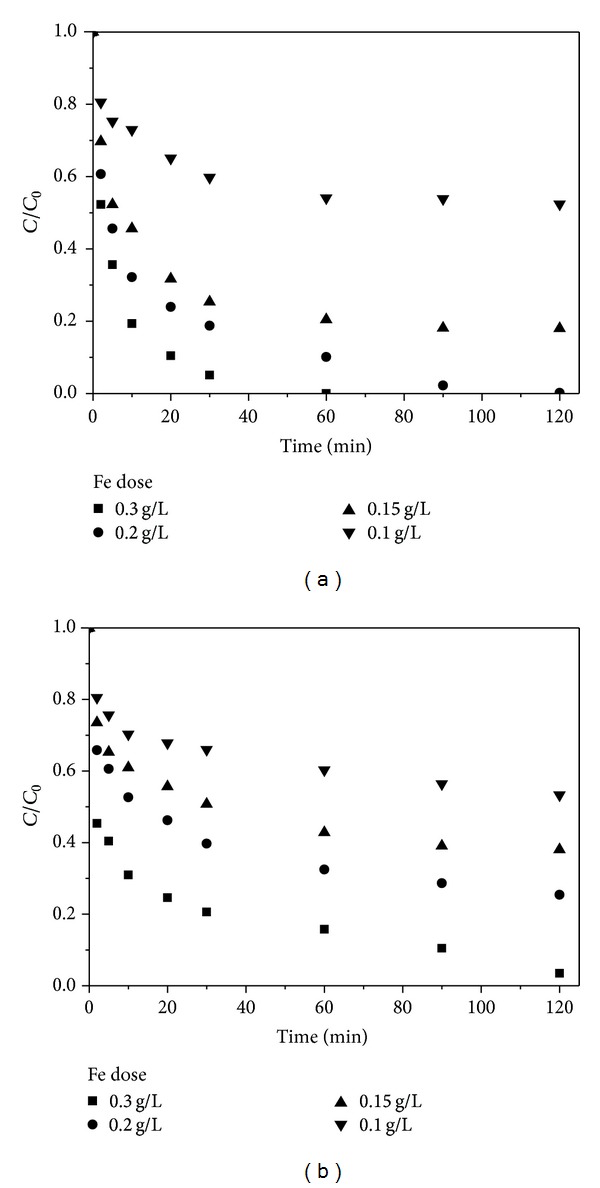
Effect of Fe NPs dosage on Cr(VI) (a) and Cd(II) (b) removal by Fe@SiO_2_. Initial Cr(VI) and Cd(II) concentration: 70 mg·L^−1^, pH: 6.0 ± 0.1, and temperature: 25°C.

The influence of Fe NPs dose on the removal of 70 mg·L^−1^ Cr(VI) and 70 mg·L^−1^ Cd(II) ions by Fe@SiO_2_ was investigated. As shown in Figure 4, the Fe NPs dose strongly affected the metal removal efficiency. When Fe dosage increased from 0.1 to 0.2 g·L^−1^, the removal rate of Cr(VI) increased from 48% to 100%, and Cd(II) removal rate increased from 47% to 75%. When Fe dosage reached 0.3 g·L^−1^, two metal ions were completely removed. This was on account of the greater surface area and the availability of more active sites at higher dosages of Fe.

### 3.5. Effect of Initial Concentration of Cr(VI) and Cd(II)

**Figure 5:**
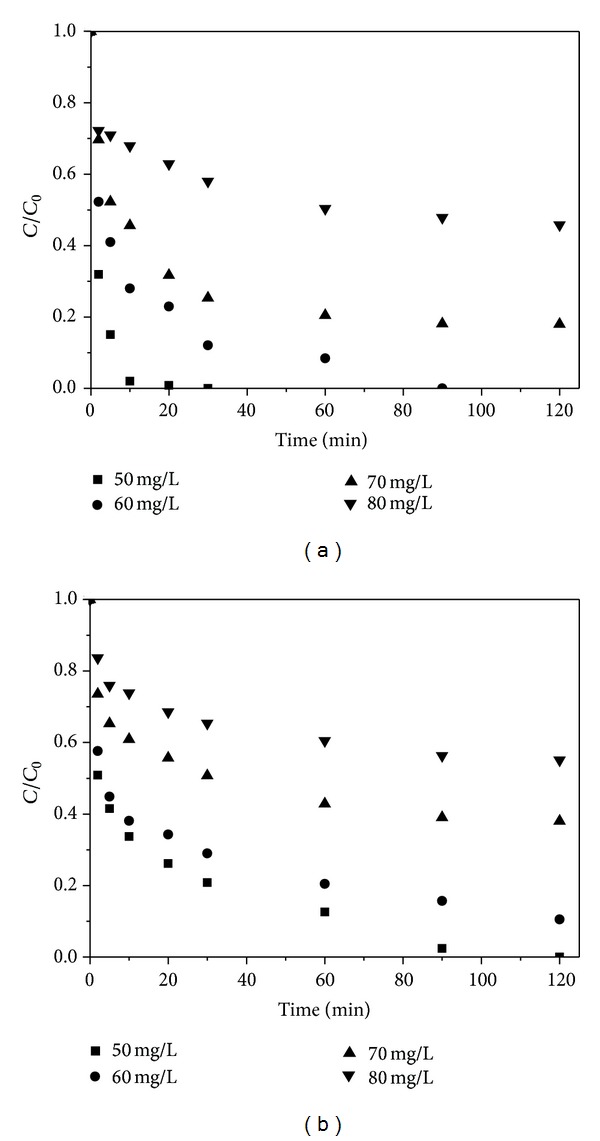
Effect of initial metal ion concentration on Cr(VI) (a) and Cd(II) (b) removal by Fe@SiO_2_. Fe dose: 0.15 g·L^−1^, pH: 6.0 ± 0.1, and temperature: 25°C.

The effect of initial Cr(VI) and Cd(II) concentrations which ranged from 50 to 80 mg·L^−1^ on degradation rates by 0.15 g·L^−1^ Fe@SiO_2_ at pH 6 was studied. As shown in Figure 5, the removal rate for both ions decreased as the initial concentrations increased. With the increase in initial ions concentration from 50 to 80 mg·L^−1^, the Cr(VI) removal capacity decreased from 100% to 54%, and the Cd(II) removal capacity decreased from 100% to 45%, respectively. This was attributed to the fact that the removals of both Cr(VI) and Cd(II) were a surface-mediated process [38]. At the lower ion concentration, the available active sites were sufficient, and thus, complete removal occurred. However, when more heavy metal ions approached the Fe^0^ surface, the available active sites for per mole ions decreased. A quickly formed passivation layer on the surface of nZVI would lead to the degeneration of reactivity [33]. On the other hand, compared to Cr(VI), a lower removal rate of Cd(II) was obtained at the same reaction condition, which was consistent with the previous section. For instance, when the initial concentrations of two metal ions were 60 mg·L^−1^, 100% Cr(VI) and 90% Cd(II) were removed, respectively.

### 3.6. Effect of pH

**Figure 6:**
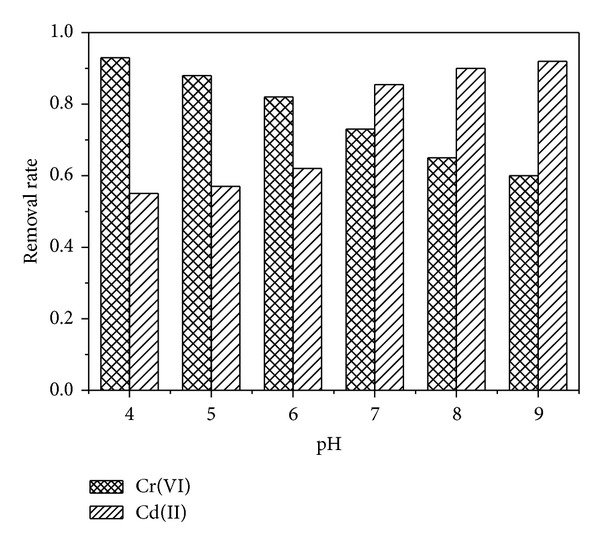
Effect of initial pH on Cr(VI) and Cd(II) removal by Fe@SiO_2_. Initial Cr(VI) and Cd(II) concentration: 70 mg·L^−1^, Fe dose: 0.15 g·L^−1^, and temperature: 25°C.

The effect of initial pH on 70 mg·L^−1^ Cr(VI) and Cd(II) removal using 0.15 g·L^−1^ Fe@SiO_2_ under temperature of 25°C was shown in Figure 6. No acid or alkali was added to maintain pH throughout the process. After reaction for 120 min, the final pH was all about 9.2 as a result of the OH^−^ release.

As shown in Figure 6, the Cr(VI) removal using Fe@SiO_2_ was much slower at high pH condition. The Cr(VI) removal rate was reduced by 33% with the pH rising from 4 to 9. According to

(5) Cr2O72−+2Fe0+7H2O  →2Cr3++14OH−+2Fe3+  

the increase of pH can decelerate the reaction rate of iron. And the high pH also can accelerate the formation of Fe(III)-Cr(III) precipitates on Fe surface, which lowers the reducing power of Fe NPs. Thus, Cr(VI) removal rate decreased with an increase in pH value.

Although chemisorption between Cd(II) and Fe surface was likely to be the major mechanism for Cd(II) adsorption, solution pH strongly influenced the Cd(II) adsorption processas indicated in Figure 6. When pH was 5, the Cd(II) adsorption on Fe@SiO_2_ was 57%. A growth adsorption was observed as pH increased. While Cd(II) removal rate reached 90% in 120 min, pH was greater than 7. This was because the solution pH affected the surface charge of Fe@SiO_2_ and the degree of ionization, as well as the speciation of the metal contaminant. At pH below the isoelectric point, the adsorbent surface was protonated, and an electrostatic repulsion existed between the positively charged surface and Cd(II) ions, resulting in the reduced Cd(II) adsorption. In addition, in the highly acidic medium, the high concentration of H^+^ ions in the solution may compete with Cd(II) for the adsorption sites, inhibiting the adsorption. At pH above the isoelectric point, the Fe@SiO_2_ surface was negatively charged, making the surface electrostatically favorable for adsorption of positive Cd(II). In the present study, the isoelectric point of Fe@SiO_2_ was about 5.2 because the Fe NPs was coated by a highly negative silica shell. At pH of 6.0, the final Cd(II) removal rate by Fe@SiO_2_ was 62%. However, only 5% Cd(II) adsorption on bare Fe NPs was obtained at pH of 6.0 as reported by Boparai et al. [32]. This was because the bare Fe NPs surface was positively charged at pH of 6. Moreover, with the simultaneous removal of Cr(VI) by Fe@SiO_2_, the solution pH immediately increased to about 9.2 and thus promoted cadmium hydrolysis/precipitation (i.e., CdOH^+^, Cd_2_(OH)^3+^, Cd(OH)_2_^0^, Cd(OH)_3_^−^, and Cd(OH)_4_^2−^). Therefore, most of the Cd(II) was adsorbed on Fe@SiO_2_ event at low initial pH. Anyway, this core-shell Fe@SiO_2_ showed a high activity in a broad range of pH which was very suitable for the practical application.

### 3.7. Effects of Interfering Substance

**Figure 7:**
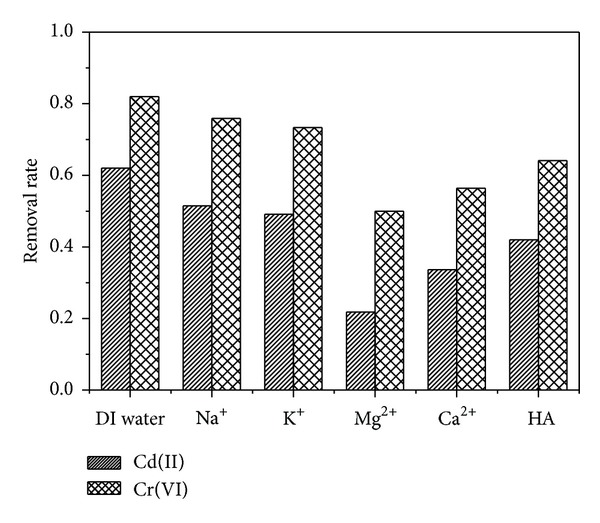
Impact of interfering substance on Cr(VI) and Cd(II) removal capacity by Fe@SiO_2_. Initial Cr(VI) and Cd(II) concentration: 70 mg·L^−1^, the Fe dose: 0.15 g·L^−1^, pH = 6.0 ± 0.1, and temperature: 25°C.

Alkali and alkaline-earth metal cations such as Na^+^, K^+^, Mg^2+^, and Ca^2+^ are commonly present with metal contaminants in polluted waters. Thus, it was necessary to study the effects of these metal ions on Cr(VI) and Cd(II) removal by Fe@SiO_2_. Meanwhile, the effect of natural organic matter (HA) on Cr(VI) and Cd(II) removal was also investigated. A sample containing a mixture of 70 mg*·*L^−1^ Cr(VI) and Cd(II) with interfering substance was prepared at pH of 6 and 25°C. As shown in Figure 7, compared with DI water, the Cr(VI) removal ability was reduced by 6.1%, 8.61%, 32.02%, 25.63%, and 17.87% in the presence of 10 mmol·L^−1^ Na^+^, K^+^, Mg^2+^, Ca^2+^, and 10 mg·L^−1^ HA, respectively. Compared with DI water, the Cd(II) removal ability was reduced by 10.5%, 12.38%, 40.2%, 28.37%, and 20.05% in the presence of 10 mmol·L^−1^ Na^+^, K^+^, Mg^2+^, Ca^2+^, and 10 mg·L^−1^ HA, respectively. It was apparent that divalent metal ions exerted more impact than monovalent metal ions on the Cr(VI) and Cd(II) removals. This was attributable to two effects: divalent metal ions can effectively compete for sorption sites and decreased the electrostatic repulsions, resulting in serious aggregation of Fe@SiO_2_ [39]. The deterioration effect of HA may be in that the adsorbed HA on silica surface can decrease the active sites of Fe NPs.

## 4. Conclusions

In conclusion, a simple method of synthesizing uniform SiO_2_-coated Fe nanoparticles (Fe@SiO_2_) can be established in a one-pot system. The resultant SiO_2_ shell not only suppressed the growth of the Fe NPs but also prevented it from aggregation. And the removals of Cr(VI) and Cd(II) by Fe@SiO_2_ under ambient condition were evaluated. The result showed that Cd(II) was adsorbed on Fe surface while Cr(VI) was reduced to Cr(III). Further, the removal rate rose as the initial concentrations of heavy metals decreased and Fe dose increased. The pH had complex effect on the Cr(VI) and Cd(II) removals by Fe@SiO_2_. The acidity of system had been found to play a major role in the reduction of Cr(VI). However, Cd(II) adsorption increased with the increased solution pH. The presence of alkali and alkaline-earth metal cations had effect on the Cr(VI) and Cd(II) removals. Summarily, the core-shell Fe@SiO_2_ may be considered as an effective material for the removals of Cr(VI) and Cd(II) from aqueous solutions.
